# Advanced Drug Delivery Micro- and Nanosystems for Cardiovascular Diseases

**DOI:** 10.3390/molecules27185843

**Published:** 2022-09-09

**Authors:** Siavash Iravani, Rajender S. Varma

**Affiliations:** 1 Faculty of Pharmacy and Pharmaceutical Sciences, Isfahan University of Medical Sciences, Isfahan 81746-73461, Iran; 2 Regional Centre of Advanced Technologies and Materials, Czech Advanced Technology and Research Institute, Palacký University in Olomouc, Šlechtitelů 27, Olomouc 78371, Czech Republic

**Keywords:** cardiovascular diseases, drug delivery nanosystems, biocompatibility, targeted drug delivery, advanced delivery systems

## Abstract

Advanced drug delivery micro- and nanosystems have been widely explored due to their appealing specificity/selectivity, biodegradability, biocompatibility, and low toxicity. They can be applied for the targeted delivery of pharmaceuticals, with the benefits of good biocompatibility/stability, non-immunogenicity, large surface area, high drug loading capacity, and low leakage of drugs. Cardiovascular diseases, as one of the primary mortalities cause worldwide with significant impacts on the quality of patients’ life, comprise a variety of heart and circulatory system pathologies, such as peripheral vascular diseases, myocardial infarction, heart failure, and coronary artery diseases. Designing novel micro- and nanosystems with suitable targeting properties and smart release behaviors can help circumvent crucial challenges of the tolerability, low stability, high toxicity, and possible side- and off-target effects of conventional drug delivery routes. To overcome different challenging issues, namely physiological barriers, low efficiency of drugs, and possible adverse side effects, various biomaterials-mediated drug delivery systems have been formulated with reduced toxicity, improved pharmacokinetics, high bioavailability, sustained release behavior, and enhanced therapeutic efficacy for targeted therapy of cardiovascular diseases. Despite the existing drug delivery systems encompassing a variety of biomaterials for treating cardiovascular diseases, the number of formulations currently approved for clinical use is limited due to the regulatory and experimental obstacles. Herein, the most recent advancements in drug delivery micro- and nanosystems designed from different biomaterials for the treatment of cardiovascular diseases are deliberated, with a focus on the important challenges and future perspectives.

## 1. Introduction

**Figure 1:**
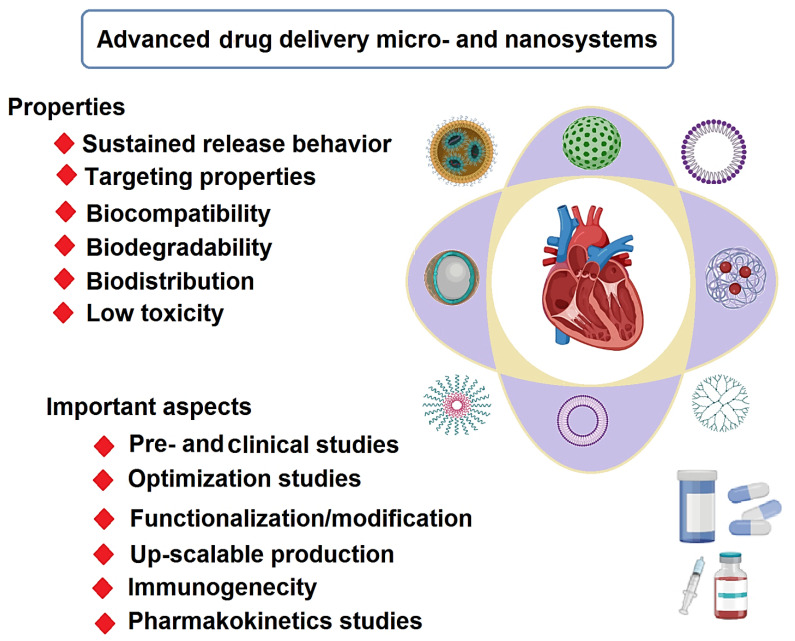
Advanced drug delivery micro- and nanosystems for CVDs: properties and challenging issues.

One of the areas of interest in nanomedicine is the design of novel targeted drug delivery systems (DDSs) with high specificity/selectivity, biodegradability, biocompatibility, and low toxicity [1,2,3,4]. Cardiovascular diseases (CVDs), as one of the primary mortalities causes worldwide with significant impacts on the quality of patients’ life, comprise a variety of heart and circulatory system pathologies, such as peripheral vascular diseases, myocardial infarction (MI), heart failure, and coronary artery diseases [5,6,7,8]. Generally, conventional synthetic drugs and natural products (e.g., curcumin, garlic, and rapamycin) have been widely employed for treating various CVDs, but they typically suffer from disadvantages of adverse side effects, low sensitivity/selectivity, low efficiency/bioavailability, and tolerance [9,10,11,12,13]. With the advancement of novel DDSs with excellent targeting properties, biodegradability, biocompatibility, and low toxicity, scientists are designing biomaterials-mediated drug delivery micro-nano systems with the benefits of good biocompatibility/stability, none-immunogenicity, large surface area, high drug loading capacity, and low leakage of drugs (Figure 1) [12,14,15,16]. To improve the bioavailability, solubility, and drug loading capacity, micro- and nanoscale DDSs have been designed with sustained and controlled release behavior for poorly soluble pharmaceuticals [17,18]. For instance, to improve the pharmacological properties of carvedilol (e.g., water solubility and bioavailability), a novel DDS was developed using halloysite nanotube capsulated in a pH-sensitive gelatin-based microsphere. This nanosystem exhibited rapid and pH-responsive drug release behavior under acidic conditions (in vitro), representing non-toxic DDS for oral drug delivery in CVDs [17]. Additionally, novel systems were developed using poly(lactide) polycarboxybetaine, cardiac homing peptide, and gold (Au) nanoparticles (NPs) to improve myocardial hypertrophy and fibrosis [19]. Overall, targeted micro- or nano-delivery of therapeutic agents represents a novel tactic in the treatment of CVDs to efficiently reduce the burden of atherosclerosis, recover the outcomes in patients with ischemic stroke, and improve the ventricular function in patients with MI and heart failure (Table 1) [20].

There are numerous innovatively designed micro- and nanosystems with diagnosis and therapeutic potentials in various forms of implants, nanorobotics, real-time monitoring systems/devices, nano-/microneedles, nanoblades, combinational therapeutic systems, among others [27,28]. Micro- and nanostructures with their unique physiochemical properties and architectures can be loaded with various therapeutic agents to show improved pharmacokinetics, pharmacodynamics, solubility, efficacy, and selectivity properties suitable for smart targeted drug delivery in treating CVDs [29,30]; the tolerability, stability, and safety of drugs can be enhanced while their toxicity and off-target properties are reduced by applying DDSs [31]. Additionally, other important criteria, such as size/morphology, surface chemistry/charge, immune responses, drug-loading content/efficacy, pharmacokinetics, surface functionalization, bioavailability, and biodegradability, ought to be considered for ensuring the safe and targeted delivery of therapeutic agents [30,32]. Notably, innovatively designed systems inspired from natural biological systems can help address some important clinical barriers, including cytotoxicity, valve thrombus, endothelialization complexity, rapid clearance, and immune responses [33]. For instance, heart valves cross-networked with erythrocyte membrane drug- filled NPs have been designed from poly(lactic-co-glycolic acid) NPs for anti-calcification, endothelialization, anticoagulation, and anti-inflammation activities. The modified valves exhibited tumor necrosis factor (TNF)-α reduction and interleukin (IL)-10 enhancement results revealed superb applicability for valvular heart disease [33]. This manuscript highlighted most recent advancements in drug delivery micro- and nanosystems designed from various biomaterials for the treatment of cardiovascular diseases, focusing on important challenges and future perspectives.

## 2. Biomaterials

### 2.1. Natural Biomaterials

Various natural polysaccharide- and protein-based (nano)structures have been utilized in developing smart DDSs for CVDs therapy purposes. Among them, chitosan-based biomaterials with biocompatibility, biodegradability, and versatility advantages have been explored for designing DDSs in cardiac therapies [34,35,36,37,38]. Assorted forms of chitosan-based materials have been prepared, including nano-coatings, microcapsules, three-dimensional-printed materials, and nanofibrous patches or scaffolds, etc. [39,40,41]. The short processing time to prepare these biomaterials, with alluring capabilities, on industrial scales makes them promising candidates for drug delivery in CVDs [42]. Cardiac extracellular matrix-chitosan-gelatin scaffolds, chitosan/dextran/*β*-glycerophosphate injectable hydrogels, chitosan/silk fibroin-modified cellulose nanofibrous patches, chitosan-gelatin based systems loaded with therapeutic/functional agents (e.g., ferulic acid), and alginate- or collagen-chitosan hydrogels are some of these systems formulated for improving vascularization, heart function, cell survival/proliferation, cell delivery for MI therapy, and expression of vascular endothelial growth factor, in addition to the sustained release of therapeutic agents [43,44,45,46]. However, there are some persistent challenges, especially pertaining to the environmentally unfriendly techniques applied for the chemical production of these materials; large amounts of alkaline wastes; or organic materials typically ensue [47].

In order to reduce the severe side effects of milrinone and improve its circulation time, an albumin protein-based nanoformulation conjugated with angiotensin II peptide (the targeting ligand) was designed for heart-targeted transferring of milrinone to improve the myocardial contractility and heart function (in vivo) [48]. Compared to the control non-targeted drug, milrinone lactate—the nanosystem exhibited—improved pharmacokinetics with efficient function for elevating the cardiac output factors. These nanosystems could also highly improve the fractional shortening factors and ejection fraction, thereby enhancing the cardiac function [48]. In addition, to treat cardiovascular pathologies, an innovatively designed poly-*L*-arginine/dextran multi-incrusted CaCO_3_-centered nanosystem was formulated with good biocompatibility and hemocompatibility, of which the endothelial cells could uptake. No noticeable induction of matrix metallopeptidase 9 (MMP-9) expression could be detected, offering nanosystems with high therapeutic efficacy in CVDs [49].

### 2.2. Synthetic Biomaterials

Synthetic biodegradable polymeric (nano)materials have been broadly explored for constructing smart micro- and nanosystems for drug delivery in treating CVDs, with biodegradability and biocompatibility advantages [50]. Hardy et al. [51] constructed a multifunctional nanosystem with high loading capacity and sustained release behavior from poly(glycidyl methacrylate) NPs and antioxidants (curcumin or resveratrol) for the transport of a peptide against the *L*-type Ca^2+^ channel to simultaneously reduce the cardiac ischemia–reperfusion injury [51]. In addition, pitavastatin could be transferred by a DDS composed of poly(lactic-co-glycolic acid) NPs to inhibit the destabilization and rupture of atherosclerotic plaque in mice via the regulation of the enrollment of inflammatory monocytes, thus providing enormous opportunities for the prevention of acute MI [52]. In addition, dipyridamole-loaded biodegradable polylactide nanoplatforms were fabricated via electrospinning deposition technique, with advantages of cost-effectiveness and sustained/controllable release behavior as coatings for cardiovascular stents. Thus, these nanosystems could be further designed with cytocompatibility advantage for antithrombotic and anti-atherosclerosis appliances [53].

Therapeutic angiogenesis can play important roles in atherosclerosis and cardiac ischemic disease by creating new blood vessels, offering the auto-rhythmicity and contractility of remaining cardiomyocytes, constraining cardiac remodeling, and stimulating infarction remedial. In this context, nanomaterials can be applied for the regulation of endothelial behavior to promote angiogenesis [54]. In one study, a DDS with no noticeable toxicity was designed from poly (lactic-co-glycolic acid) NPs comprising adrenomedullin-2 for therapeutic angiogenesis. Accordingly, adrenomedullin-2 was sustained released from this nanosystem for ~21 days for the induction of cell proliferation in endothelial cells (in vitro), demonstrating the attractive angiogenic peptide delivery property of this nanosystem for therapeutic angiogenesis purposes in CVDs via growth factor-based therapeutic strategies [55]. In addition, to solve the restricted targeting properties and rapid clearance of routinely applied drugs for atherosclerosis, core-shell nanosystems with improved targeting property and multifunctionality have been formulated using ginsenoside- and catalase-co-loaded porous poly(lactic-co-glycolic acid) NPs, after the additional surface functionalization with U937 cell membranes [56]. These nanosystems could escape from phagocytosis of macrophages and specifically target atherosclerotic plaques; suitable antioxidant activities and H_2_O_2_-responsive drug release, as well as reactive oxygen scavenging properties and down-regulation of some factors (e.g., IL-1β, TNF-α, and ICAM-1) could be attained due to the loading with catalase and bioactive agents [56].

Biodegradable porous silicon NPs were functionalized with atrial natriuretic peptide A for directed drug transport into the endocardial layer of the left ventricle with the purpose of cardiac therapy. The prepared system exhibited improved cellular exchanges with cardiomyocytes and non-myocytes in addition to the enhanced colloidal stability with no noticeable toxicity, showing cardio-protective potentials (particularly ischemic heart disease) [57]. Additionally, the amelioration of angiogenesis and cardiac operation in infarcted heart tissue was reported via local transport of exosomes using antibody-conjugated magnetic NPs comprising Fe_3_O_4_ (core) and silica (shell) NPs adorned with poly (ethylene glycol). These nanosystems could successfully target CD63 antigens on the surfaces of extracellular vesicles or myosin-light-chain surface markers on damaged cardiomyocytes [58]; exosomes are associated with cardiac myocytes, as well as stem, progenitor, endothelial, and vascular cells, paying crucial therapeutic and diagnostic roles in CVDs (especially cardiac regeneration) [59].

## 3. Drug Delivery Micro-/Nanosystems for CVDs

### 3.1. Hydrogels

**Figure 2:**
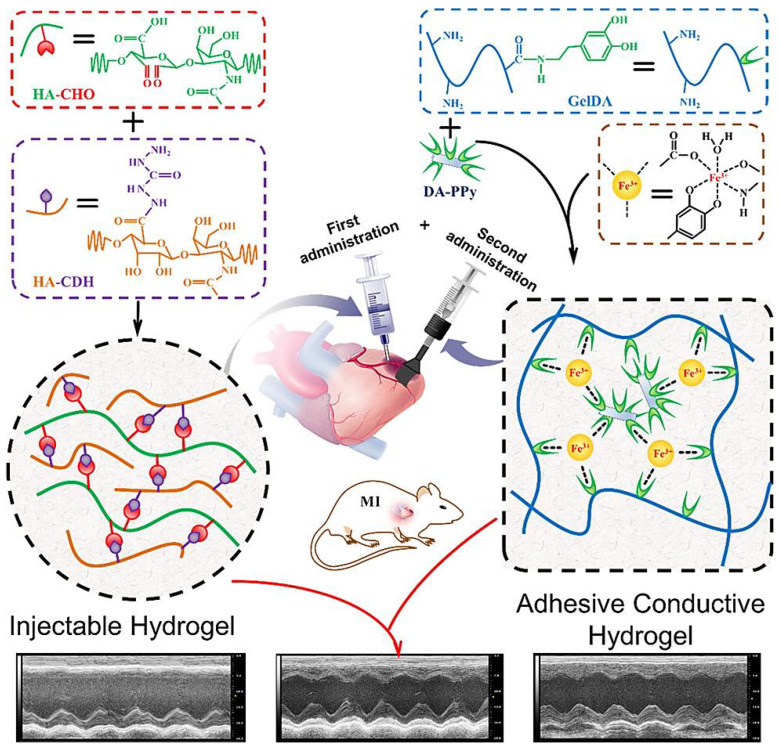
The design of an injectable hydrogel and adhesive hydrogel patch, as well as the combined internal–external treatment strategy for MI. HA-CHO: oxidized sodium hyaluronic acid; HHA: hydrazided hyaluronic acid; GelDA: dopamine-gelatin; DA–PPy: dopamine-functionalized polypyrrole. Adapted from Ref. [62] with permission. Copyright 2019 American Chemical Society.

Various types of functional hydrogels with attributes of biocompatibility, controllable swelling behavior, and biodegradability have been widely formulated using biomaterials (e.g., chitosan, heparin, fibrin, collagen, gelatin, etc.) with the purpose of MI therapy [60,61,62]. Based on their functions and properties, these hydrogels have been categorized as matrix metalloproteinase (MMP)-responsive, immunomodulatory, conductive, proangiogenic, three-dimensional printed hydrogels, among others [60,63,64]. For instance, a conductive and adhesive hydrogel without adverse liquid leakage was designed as a therapeutic cardiac patch centered on Fe^3+^-initiated concurrent polymerization of covalently connected dopamine and pyrrole to improve the cardiac function reconstruction, as well as the infarct myocardium revascularization [65]. In addition, a self-adhesive conductive hydrogel patch was designed based on Fe^3+^-stimulated ionic coordination between dopamine-gelatin conjugates and dopamine-functionalized polypyrrole, which formed a homogeneous network. The injectable and cleavable hydrogel was prepared (in situ) through a Schiff base reaction between oxidized sodium hyaluronic acid and hydrazided hyaluronic acid. The adhesive conductive hydrogel patch together with an injectable hydrogel could achieve improvement of the cardiac function, providing efficient hydrogel-based systems for treating MI (Figure 2) [62]. For mesenchymal stromal cell-based therapy after MIs, several peptide-crosslinked polyethylene glycol-based micro-/nanosystems were designed with advantages of inherent hydrophilicity, easy functionalization with bioactive peptides, and protein-resistance properties, thus enabling the adjustment of cell-based degradability and promoting cell adhesion [66]. However, they may suffer from the possible immune reactions (repeated sensitization), restricted degrees of functionalization, and enough flexibility in structural design.

Several explorations have been conducted on designing micro-/nanosystems loaded with cardiac stem cells (CSCs) in CVDs. As an example, vascularized cardiac patches containing biomimetic microvessels encapsulated in a fibrin gel spiked with human CSCs were constructed for promoting the proliferation of cardiomyocytes and neovascularization after acute MI, providing vast opportunities for treating ischemic heart injuries [67]. In one study, Hua et al. [68] reported an injectable hydrogel constructed from chitosan, dextran, and *β*-glycerophosphate loaded with human mesenchymal stem cells for cardiac healing after acute MI. The designed nanosystem exhibited significant stability with unique physicochemical properties suitable for cardiac regeneration appliances. After in vitro evaluations, it was established that the cell survival rate was improved and the expression of pro-inflammatory factors was increased. The analysis of pro-angiogenic factors illustrated promising outcomes regarding the fibrosis area, vessel density with reduced size of infractions, and ejection fraction, providing superb opportunities for healing cardiac function after MIs [68]. Impressively, biodegradable poly(2-alkyl-2-oxazoline) hydrogels with high tissue adhesive features and advantages of rapid healing property (<2 min) after photo-irradiation, owing to the di-cysteine cell degradable peptides, could enable the cell protrusion regulation in three-dimensional matrices, regulating the secretory phenotype of mesenchymal stromal cells [69]. These bioactive hydrogels with controlled mechanical properties could stimulate the regaining of cardiac function/structure including recovered neovascular generation and decreased interstitial fibrosis, in vivo. Notably, cell-degradable and mechanical features of these hydrogels could be enhanced to adjust the secretory phenotype of mesenchymal stromal cells, especially for accelerating the secretion of cytokines and stimulating growth factors for additional vascularization [69].

### 3.2. Liposomes

**Figure 3:**
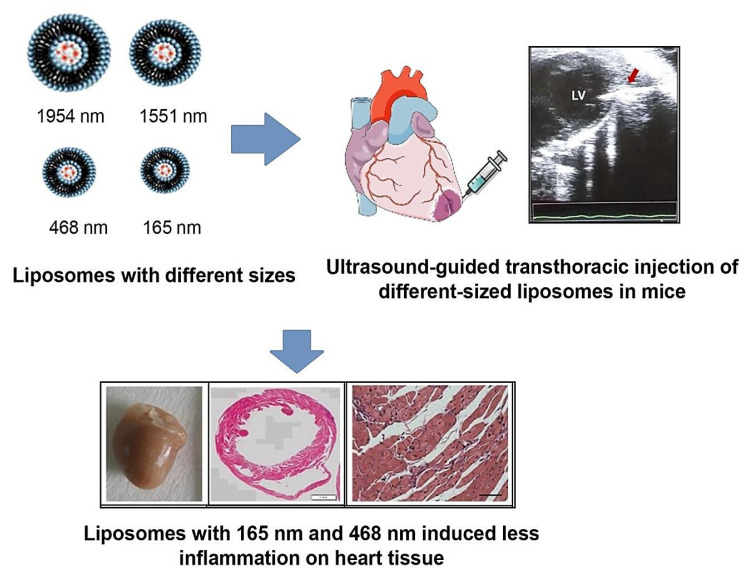
Liposome-based systems for targeted delivery of biopharmaceuticals in heart diseases, with related inflammation on heart tissue. Adapted from Ref. [75] with permission. Copyright 2022 Elsevier.

Liposomes with unique physicochemical and biophysical characteristics have shown suitable biocompatibility, self-assembly potentials, high drug loading, and encapsulating capacity, and their controllable/sustained release behavior can be considered for designing smart DDSs in treating CVDs [70]. Various target-specific liposomes have been developed for the delivery of small molecule drugs in CVDs, especially after reperfused MI. For instance, specific peptides with high affinity to the cells existed in the post-infarct myocardium (e.g., endothelial cells, myofibroblasts, and cardiomyocytes) were conjugated with liposomes for delivering poly [ADP-ribose] polymerase 1 inhibitor, demonstrating an efficient DDS for targeted therapy in the infarct border zone [71]. Furthermore, microRNA-21 with responsibility to the pathophysiological effects of acute MI by affecting downstream vital regulators was encapsulated into liposome functionalized with the cardiac troponin T antibody for specific transfer to the ischemic myocardium, thus resolving the inadequate cellular uptake and poor stability challenges [72]. The nanosystem exhibited improved targeting features to hypoxia primary cardiomyocytes (in vitro) and enhanced accumulations in the ischemic heart of rats with acute MI after injecting in the tail vein because of specific targeting to the overexpressed troponin, consequently improving the cardiac function and decreasing the infarct size, together with the viability maintenance in cardiomyocytes [72]. Despite the clinical potentials of these formulations, several important challenges and pitfalls persist, namely the possible interactions of liposomes with the immune system, as well as the possibility of antibody formation against surface-functionalized/modified liposomes as various components or encapsulated cargos may restrict their clinical translation [73,74]. Santos et al. [75] developed liposome-based systems constructed via the lipid thin film hydration technique followed by sonication for cardiac drug delivery (in vivo). After assessment of these nanosystems in MI induced by isoproterenol in mice, it was revealed that the cytotoxic and inflammatory effects of them were size dependent (Figure 3) [75].

### 3.3. Dendrimers

Dendrimers with drug delivery capability along with the availability of multiple functional groups can be employed for stabilizing drugs, enhancing the solubility of therapeutic agents, and improving the sustained/controllable release of drugs/bioactive agents [76,77]. For instance, dendrimer NPs loaded with simvastatin acid were designed with adsorption ability to the surface of red blood cells, providing reactive oxygen stress (ROS) and shear stress dual-sensitive bionic systems for treating atherosclerosis. Consequently, the prepared systems with sustained release behavior exhibited better therapeutic effects toward atherosclerosis and high in vivo safety [78]. Dendrimer-*N*-acetylcysteine conjugate nanosystems were developed for targeting activated microglial cells subsequent to cardiac arrest to provide improvements in survival rate, neurological recovery, and short-term motor shortfalls. These nanosystems offer as promising strategies for the treatment of post-cardiac arrest syndrome [79].

### 3.4. Niosomes

Niosomes have shown some salient advantages, compared to liposomes, including the suitable physicochemical stability, cost-effectiveness, simple formulation processes, and up-scalable potentials. They have been utilized for constructing a variety of formulations in treating CVDs. In one study, to solve the broad pre-systemic disposition and low rate of dissolution, chitosan-encapsulated niosomes were formulated to improve the oral delivery of atorvastatin, providing enhanced anti-hyperlipidemic effects [80]. Additionally, to improve the poor oral bioavailability of rosuvastatin, the niosome-based systems were developed via the film hydration technique and sonication utilizing Span 40 and cholesterol. As a result, the permeation of rosuvastatin was significantly enhanced (~95.5% after 2 h) and its oral bioavailability was improved [81]. Simvastatin-loaded nano-niosomes were designed to improve water solubility, half-life, and bioavailability of this drug against myocardial ischemia and reperfusion injury. These nanosystems could efficiently improve cardiac function and inhibit the necroptosis trail [35].

### 3.5. Solid Lipid NPs

Lipid-based nanosystems have shown alluring benefits, such as up-scalable production, biocompatibility, biodegradability, low toxicity, controllable/sustained drug release behavior, and possibility of drug solubility improvements [82]. For instance, daidzein-loaded solid lipid NPs with sustained release behavior, improved the pharmacokinetics, and significant increase in circulation time can be employed for treating cardio-cerebrovascular diseases [83]. In addition, solid lipid nanosystems were designed for the delivery of phytostanols for the management of hypercholesterolemia, signifying the potentials of these NPs in treating CVDs [84]. However, limited studies have been focused on this field of science, and more elaborative works ought to be planned for constructing novel solid lipid NPs for CVDs therapy.

### 3.6. Nanocapsules

Nanocapsules have been widely prepared for controlled drug release to specifically target the sites with a variety of drugs, bioactive agents, and protein/peptide compounds [85]. Chaves et al. [86] introduced nanocapsules for improving oral bioavailability of carvedilol. These nanosystems exhibited controlled drug release behavior with mucoadhesive features and enhanced retention time, offering suitable sublingual dosage forms in treating CVDs [86]. Further, shear-sensitive nanocapsules have been developed for the site-specific drug release with inhibitory effects against occlusive thrombus generation. Consequently, thrombus generation was selectively inhibited (in vitro) under stenotic and excessive shear flow circumstances. Future explorations need to focus on in vivo and clinical applicability of these nanosystems [87].

## 4. Cardiovascular Organ-on-Chip Platforms

Organ-on-chip platforms comprising microfluidics, cell tissue/organ, stimulation, and sensor systems can be applied in the development and discovery of new drugs for treating CVDs due to their excellent potentials in achieving a physiological resemblance in vitro, offering new opportunities to evaluate the efficiency and toxicity of drugs and study the cardiovascular pathophysiology instead of using traditional in vitro cell cultures [88,89,90]. In addition, these platforms can be employed for evaluating the progression of CVDs (e.g., heart-on-chip and blood vessels-on-a-chip). Notably, the cardiotoxicity induced by drugs is one of the important concerns in drug development pipelines [91,92]. However, the routinely applied in vitro and in vivo assessments may suffer from higher costs, lack of reliability/accuracy in cardiotoxicity prediction, and inter-species differences. Microfluidic heart-on-chip devices can recapitulate the functionality levels of cardiac tissue and the communication between cells and extracellular matrices, thus providing suitable platforms for measurement of cellular dynamics along with the computational modeling for clinical purposes [91,93]. Moreover, they can be applied for drug analysis/monitoring due to their excellent functionality and sensing capabilities to assess the disease-specific phenotypic, genotypic, and electrophysiological details in real-time. The electrophysiology and mechanobiology of the evaluations can be used for better mimicking the in vivo conditions using these platforms with unique electrical and mechanical properties [93,94]. In one study, a cardiac chip was designed for recording cardiac tissue adhesion, electrophysiology, and contractility [95]. This platform applied the transplantation of cardiomyocytes derived from human-induced pluripotent stem cells to evaluate the electrophysiology and contractility of myocardial cells under physiological conditions and drug stimulation, respectively. In addition, it was deployed for testing the effects of norepinephrine clinically deployed for the treatment of hypotension and heart failure. The electrical stimulation using micro-fabricated electrodes could highly improve the structure and arrangement of cardiac cells [95]. Furthermore, a heart/liver-on-a-chip was developed using the HepG2 hepatocellular carcinoma cells and H9c2 rat cardiomyocytes to reproduce the cardiotoxicity stimulated by doxorubicin (in vitro) [96]. As a result, more significant damage to heart cells could be detected within the heart/liver-on-a-chip than conventional static three-dimensional culture used for cancer therapy by doxorubicin, because of the disclosure of cells to both the main drug and its cardio-toxic metabolites (doxorubicinol) [96]. By designing novel organ-on-chip platforms, the currently applied preclinical assessments can be improved; moreover, the efficiency and possible off-target toxicity/side effects of micro-/nanosystems designed for the treatment of CVDs can be better evaluated [97]. However, several challenges regarding the vascularization of tissues, controlling the cell density, reproducibility, cell viability assessments, cell–cell interactions monitoring, suitable organ scaling, sterility maintenance, incorporation of cultured organoids, and sensing modules, among others. [90].

Generally, several materials have been explored for manufacturing different organ-on-chip platforms, such as poly(dimethylsiloxane) and poly(methyl methacrylate) [98,99]. Some other materials, such as poly(acrylic acid), have been deployed for promoting the adhesion between the two parts; polycarbonate, poly(ethylene terephthalate), and polystyrene were also utilized for improving the connection between the poly (methyl methacrylate) substrates [100]. Overall, poly(dimethylsiloxane) exhibited some advantages, such as low cost, easy-to-manufacture, optical transparency, biocompatibility, non-toxicity, permeability to gases, chemical inertness, thermal stability, ultraviolet resistance, and no treatment required for long-term storage. However, it may suffer from some limitations, including non-specific absorption of molecules or incompatibility with specific reagents. In addition, hydrogels have been applied for manufacturing organ-on-chip platforms, with advantages of cost-effectiveness and biocompatibility but with some drawbacks of weak mechanical strength and the need for freezing/drying for long-term storage [98]. In one study, a blood vessel/tissue model was developed utilizing poly(methyl methacrylate) and poly(ethylene terephthalate) to analyze the procedure of leucocyte infiltration, in addition to their possible interactions with macrophages from the blood vessel [101]. Notably, an ultrathin layer of polycaprolactone was electrospun on the poly(methyl methacrylate) substrate. The endothelial cells were seeded to recreate the blood vessel in one side of the membrane and also a collagen gel layer integrated with macrophages was located on the other side, thus offering a promising model for observing leucocyte infiltration and interaction with perivascular macrophages [101]. In addition, Annabi et al. [102] introduced a technique for coating microfluidic channels inside a closed poly(dimethylsiloxane) device with a cell-compatible hydrogel layer; these tropoelastin-based hydrogels can be applied as coating materials for organ-on-chip purposes [102].

## 5. Clinical Studies

One of the most important challenging issues in designing advanced DDSs for treating CVDs is clinical trials/studies. Since limited studies have been introduced on micro-/nanosystems for targeted drug delivery in CVDs, future explorations ought to focus on this crucial step [103,104,105]. There are some patents describing the therapeutic cardiac patches, such as the ultraviolet-crosslinkable gelatin methacrylate-based cardiac patch with high surface area and electrical conductivity, which was filled with gold nanorods (US20170143871 A1). Moreover, solid lipid NPs with clinical potentials could be applied for treating coronary heart disease (CN103027981B). However, only a few of these explorations/patents have entered into clinical trials because of the stringent regulatory necessities [103,104]. As an example, a study was designed to evaluate the effects of liposomal glucocorticoids administered intravenously in patients with an increased risk of atherosclerotic disease, aiming to reduce vessel wall inflammation (NCT01601106); additional clinical trials/studies can be found at https://clinicaltrials.gov/ (9 August 2022).

## 6. Conclusions and Future Outlooks

To overcome several challenges, such as physiological barriers, off-target effects, low efficiency of drugs, and possible adverse side effects, various biomaterials-mediated DDSs have been formulated with reduced toxicity, improved pharmacokinetics, high bioavailability, sustained release behavior, and increased therapeutic efficacy for targeted therapy of CVDs. Despite the pervasiveness of numerous micro- and nano-DDSs comprising various biomaterials introduced for treating CVDs, the number of formulations currently approved for clinical use is rather limited due to the regulatory and experimental obstacles. Indeterminate toxicity (study on mechanisms of toxicity) and the lack of systematical analysis of these materials restrict their further applications. There is limited relevant research evidence on the biological endpoints to evaluate the relationship between the physicochemical features of micro- and nano-DDSs, such as morphology, size, size distribution, chemical/surface structure, and electrochemical features, with their inflammatory and toxic effects. Comprehensive toxicological evaluations and cost benefits of designed DDSs are essential for their future clinical applicability. In addition, several issues pertaining to the good manufacturing practice (GMP), biosafety/tolerability, stability, immune reactions, biocompatibility/biodegradability, drug clearance, drug release/loading, pharmacokinetics/pharmacodynamics, and scaling up processes should be systematically studied to translate micro- and nano-DDSs from laboratory and preclinical phases into clinical stages. In this context, smart stimuli-responsive biomaterials-mediated DDSs should be further designed that can respond to the modified environmental signals with alterations in their morphologies and physicochemical characteristics, thus offering targeted therapy opportunities. One of the most important challenges is to simultaneously improve the multifunctionality, biocompatibility, and targeting properties of micro- and nano-DDSs, which need to be explored in future.

For clinical applications of nanomedicine in treating CVDs, there are still limited studies performed. Future explorations should be planned in both pre- and clinical trial stages for designing smart micro-/nanosystems for treating atherosclerosis, thrombosis, stroke, MI, hypertension, and pulmonary arterial hypertension with improved body circulation and drug solubility, as well as reduced amount of drugs and low cytotoxicity. Notably, some grant challenges, such as understanding the related mechanisms of actions, designing smart micro-/nano-DDSs with personalized therapy purposes, improving drug bioavailability and circulation time, and evaluating clinical safety and translation, are still remained. Controlled and sustained delivery of pharmaceuticals/therapeutic agents with advantages of disease-specific treatment can reduce off-target influences by a stimuli-mediated drug release in the affected area (especially in atherosclerosis and thrombosis). In this context, cardiovascular organ-on-chip platforms can help to specifically evaluate the efficiency and possible off-target toxicity of new drugs and their metabolites, thus offering new opportunities for better pre-clinical evaluations, drug discovery/development, and personalized medicine.

## Figures and Tables

**Table 1:** Some selected examples of micro-/nanosystems for the treatment of CVDs.

Micro-/Nanosystems	Applications	Advantages/Benefits	Refs.
Liposomes containing vascular endothelial growth factor (VEGF)	To treat MI and improve cardiac function	High enhancement in fractional shortening and improvement in systolic function; excellent improvements in cardiac function and vascular structure	[21]
Polylactic co-glycolic acid NPs containing VEGF	To repair the heart after MI	Enhancement in the angiogenic and therapeutic potency of VEGF for treating ischemic heart disease.	[22]
Polymeric NPs	For the targeted delivery of nitric oxide (NO); treatment of portal hypertension	Non-toxicity; these NPs could alleviate the hemodynamic disorders in bile duct-ligated-induced portal hypertension, evidenced by reducing portal pressure and unchanging mean arterial pressure.	[23]
Niosomes	For the delivery of lacidipine; the management of hypertension	An enhancement in skin permeation (~2.15 times), compared to control gel; improved reduction in blood pressure	[24]
Nano-vesicular lipid carriers	For the delivery of angiotensin II receptor blocker (valsartan)	Improved anti-hypertensive effects; no skin toxicity	[25]
Dendrimeric NPs	For selective delivery of liver-x-receptor ligands to atherosclerotic plaque-associated macrophages while limiting hepatic uptake; modulation of atherosclerosis	High reduction in atherosclerotic plaque progression, plaque necrosis, and plaque inflammation; macrophage-specific delivery platforms for targeted transferring anti-atherosclerotic agents to the plaque-associated macrophages to reduce plaque burden	[26]

## Data Availability

Not applicable.

## References

[1] Jamalipour Soufi G., Iravani S. (2020). Eco-friendly and sustainable synthesis of biocompatible nanomaterials for diagnostic imaging: Current challenges and future perspectives. Green Chem..

[2] Sajjadi M., Nasrollahzadeh M., Jaleh B., Jamalipour Soufi G., Iravani S. (2021). Carbon-based nanomaterials for targeted cancer nanotherapy: Recent trends and future prospects. J. Drug Target..

[3] Iravani S., Varma R.S. (2021). Plant pollen grains: A move towards green drug and vaccine delivery systems. Nano-Micro Lett..

[4] Ghovvati M., Kharaziha M., Ardehali R., Annabi N. (2022). Recent Advances in Designing Electroconductive Biomaterials for Cardiac Tissue Engineering. Adv. Healthc. Mater..

[5] Timmis A., Townsend N., Gale C., Grobbee R., Maniadakis N., Flather M., Wilkins E., Wright L., Vos R., Bax J. (2018). European Society of Cardiology: Cardiovascular Disease Statistics 2017. Eur. Heart J..

[6] Timmis A., Townsend N., Gale C.P., Torbica A., Lettino M., Petersen S.E., Mossialos E.A., Maggioni A.P., Kazakiewicz D., May H.T. (2020). European Society of Cardiology: Cardiovascular Disease Statistics 2019. Eur. Heart J..

[7] Timmis A., Vardas P., Townsend N., Torbica A., Katus H., Smedt D.D., Gale C.P., Maggioni A.P., Petersen S.E., Huculeci R. (2022). European Society of Cardiology: Cardiovascular disease statistics 2021. Eur. Heart J..

[8] Azwa Zuraini N.Z., Sekar M., Wu Y.S., Gan S.H., Bonam S.R., Mat Rani N.N.I., Begum M.Y., Lum P.T., Subramaniyan V., Fuloria N.K. (2021). Promising Nutritional Fruits Against Cardiovascular Diseases: An Overview of Experimental Evidence and Understanding Their Mechanisms of Action. Vasc. Health Risk Manag..

[9] Shi H.-T., Huang Z.-H., Xu T.-Z., Sun A.-J., Ge J.-B. (2022). New diagnostic and therapeutic strategies for myocardial infarction via nanomaterials. eBioMedicine.

[10] Hajipour M.J., Mehrani M., Abbasi S.H., Amin A., Kassaian S.E., Garbern J.C., Caracciolo G., Zanganeh S., Chitsazan M., Aghaverdi H. (2019). Nanoscale Technologies for Prevention and Treatment of Heart Failure: Challenges and Opportunities. Chem. Rev..

[11] Iravani S., Jamalipour Soufi G. (2022). Algae-derived materials for tissue engineering and regenerative medicine applications: Current trends and future perspectives. Emergent Mater..

[12] Iravani S., Varma R. (2019). Plant-derived Edible Nanoparticles and miRNAs: Emerging Frontier for Therapeutics and Targeted Drug-delivery. ACS Sustain. Chem. Eng..

[13] Iravani S., Varma R.S. (2019). Plants and plant-based polymers as scaffolds for tissue engineering. Green Chem..

[14] Pala R., Anju V.T., Dyavaiah M., Busi S., Nauli S.M. (2020). Nanoparticle-Mediated Drug Delivery for the Treatment of Cardiovascular Diseases. Int. J. Nanomed..

[15] Skourtis D., Stavroulaki D., Athanasiou V., Fragouli P.G., Iatrou H. (2020). Nanostructured Polymeric, Liposomal and Other Materials to Control the Drug Delivery for Cardiovascular Diseases. Pharmaceutics.

[16] Tapeinos C., Gao H., Bauleth-Ramos T., Santos H.A. (2022). Progress in Stimuli-Responsive Biomaterials for Treating Cardiovascular and Cerebrovascular Diseases. Small.

[17] Jaberifard F., Arsalani N., Ghorbani M., Mostafavi H. (2022). Incorporating halloysite nanotube/carvedilol nanohybrids into gelatin microsphere as a novel oral pH-sensitive drug delivery system. Colloids Surf. A Physicochem. Eng. Asp..

[18] Ruiz A.L., Ramirez A., McEnnis K. (2022). Single and Multiple Stimuli-Responsive Polymer Particles for Controlled Drug Delivery. Pharmaceutics.

[19] Dong Y., Wang B., Liang T., Huang D., Jin J., Li W., Fu L. (2022). Melatonin Loaded Cardiac Homing Peptide-Functionalized Gold Nanoparticles for the Care of Anti-Cardiac Hypertrophy. J. Polym. Environ..

[20] Cicha I. (2021). The grand challenges in cardiovascular drug delivery. Front. Drug. Deliv..

[21] Scott R.C., Rosano J.M., Ivanov Z., Wang B., Chong P.L.-G., Issekutz A.C., Crabbe D.L., Kiani M.F. (2009). Targeting VEGF-encapsulated immunoliposomes to MI heart improves vascularity and cardiac function. FASEB J..

[22] Oduk Y., Zhu W., Kannappan R., Zhao M., Borovjagin A.V., Oparil S., Zhang J.J. (2018). VEGF nanoparticles repair the heart after myocardial infarction. Am. J. Physiol. Heart Circ. Physiol..

[23] Duong H.T.T., Dong Z., Su L., Boyer C., George J., Davis T.P., Wang J. (2015). The use of nanoparticles to deliver nitric oxide to hepatic stellate cells for treating liver fibrosis and portal hypertension. Small.

[24] Qumbar M., Ameeduzzafar, Imam S.S., Ali J., Ahmad J., Ali A. (2017). Formulation and optimization of lacidipine loaded niosomal gel for transdermal delivery: In-vitro characterization and in-vivo activity. Biomed. Pharmacother..

[25] Ahad A., Aqil M., Kohli K., Sultana Y., Mujeeb M. (2016). Nano vesicular lipid carriers of angiotensin II receptor blocker: Anti-hypertensive and skin toxicity study in focus. Artif. Cells Nanomed. Biotechnol..

[26] He H., Yuan Q., Bie J., Wallace R.L., Yannie P.J., Wang J., Lancina M.G.R., Zolotarskaya O.Y., Korzun W., Yang H. (2018). Development of mannose functionalized dendrimeric nanoparticles for targeted delivery to macrophages: Use of this platform to modulate atherosclerosis. Transl. Res..

[27] Jones A.D., Mi G., Webster T.J. (2019). A Status Report on FDA Approval of Medical Devices Containing Nanostructured Materials. Trends Biotechnol..

[28] Iafisco M., Alogna A., Miragoli M., Catalucci D. (2019). Cardiovascular nanomedicine: The route ahead. Nanomedicine.

[29] Choi Y.H., Han H.K. (2018). Nanomedicines: Current status and future perspectives in aspect of drug delivery and pharmacokinetics. J. Pharm. Investig..

[30] Mohamed N.A., Marei I., Crovella S., Abou-Saleh H. (2022). Recent Developments in Nanomaterials-Based Drug Delivery and Upgrading Treatment of Cardiovascular Diseases. Int. J. Mol. Sci..

[31] Wang Y., Pisapati A.V., Zhang X.F., Cheng X. (2021). Recent developments in nanomaterial-based shear-sensitive drug delivery systems. Adv. Healthc. Mater..

[32] Dormont F., Varna M., Couvreur P. (2018). Nanoplumbers: Biomaterials to fight cardiovascular diseases. Materialstoday.

[33] Hu C., Luo R., Wang Y. (2020). Heart Valves Cross-Linked with Erythrocyte Membrane Drug-Loaded Nanoparticles as a Biomimetic Strategy for Anti-coagulation, Anti-inflammation, Anti-calcification, and Endothelialization. ACS Appl. Mater. Interfaces.

[34] Deng Y., Zhang X., Shen H., He Q., Wu Z., Liao W., Yuan M. (2020). Application of the Nano-Drug Delivery System in Treatment of Cardiovascular Diseases. Front. Bioeng. Biotechnol..

[35] Naseroleslami M., Mousavi Niri N., Akbarzade I., Sharifi M., Aboutaleb N. (2022). Simvastatin-loaded nano-niosomes confer cardioprotection against myocardial ischemia/reperfusion injury. Drug Deliv. Transl. Res..

[36] Varma R.S. (2014). Journey on greener pathways: From the use of alternate energy inputs and benign reaction media to sustainable applications of nano-catalysts in synthesis and environmental remediation. Green Chem..

[37] Varma R.S. (2016). Greener and Sustainable Trends in Synthesis of Organics and Nanomaterials. ACS Sustain. Chem. Eng..

[38] Varma R.S. (2019). Biomass-Derived Renewable Carbonaceous Materials for Sustainable Chemical and Environmental Applications. ACS Sustain. Chem. Eng..

[39] Silva A.K.A., Letourneur D., Chauvierre C. (2014). Polysaccharide Nanosystems for Future Progress in Cardiovascular Pathologies. Theranostics.

[40] Ghofrani A., Taghavi L., Khalilivavdareh B., Shirvan A.R., Nouri A. (2022). Additive manufacturing and advanced functionalities of cardiac patches: A review. Eur. Polym. J..

[41] Mousa H.M., Ali M.G., Rezk A.I., Nasr E.A., Hussein K.H. (2022). Development of conductive polymeric nanofiber patches for cardiac tissue engineering application. J. Appl. Polym. Sci..

[42] Patel B., Manne R., Patel D.B., Gorityala S., Palaniappan A., Kurakula M. (2021). Chitosan as Functional Biomaterial for Designing Delivery Systems in Cardiac Therapies. Gels.

[43] Lv J., Liu W., Shi G., Zhu F., He X., Zhu Z., Chen H. (2019). Human cardiac extracellular matrix-chitosan-gelatin composite scaffold and its endothelialization. Exp. Ther. Med..

[44] Ke X., Li M., Wang X., Liang J., Wang X., Wu S., Long M., Hu C. (2020). An injectable chitosan/dextran/β -glycerophosphate hydrogel as cell delivery carrier for therapy of myocardial infarction. Carbohydr. Polym..

[45] Chen J., Zhan Y., Wang Y., Han D., Tao B., Luo Z., Ma S., Wang Q., Li X., Fan L. (2018). Chitosan/silk fibroin modified nanofibrous patches with mesenchymal stem cells prevent heart remodeling post-myocardial infarction in rats. Acta Biomater..

[46] Deng B., Shen L., Wu Y., Shen Y., Ding X., Lu S., Jia J., Qian J., Ge J. (2015). Delivery of alginate-chitosan hydrogel promotes endogenous repair and preserves cardiac function in rats with myocardial infarction. J. Biomed. Mater. Res. Part A.

[47] Jiménez-Gómez C.P., Cecilia J.A. (2020). Chitosan: A Natural Biopolymer with a Wide and Varied Range of Applications. Molecules.

[48] Lomis N., Sarfaraz Z.K., Alruwaih A., Westfall S., Shum-Tim D., Prakash S. (2021). Albumin Nanoparticle Formulation for Heart-Targeted Drug Delivery: In Vivo Assessment of Congestive Heart Failure. Pharmaceuticals.

[49] Ferrari P.F., Zattera E., Pastorino L., Perego P., Palombo D. (2021). Dextran/poly-L-arginine multi-layered CaCO_3_-based nanosystem for vascular drug delivery. Int. J. Biol. Macromol..

[50] Matoba T., Koga J.-I., Nakano K., Egashira K., Tsutsui H. (2017). Nanoparticle-mediated drug delivery system for atherosclerotic cardiovascular disease. J. Cardiol..

[51] Hardy N., Viola H.M., Johnstone V.P.A., Clemons T.D., Szappanos H.C., Singh R., Smith N.M., Iyer K.S., Hool L.C. (2015). Nanoparticle-Mediated Dual Delivery of an Antioxidant and a Peptide against the L-Type Ca^2+^ Channel Enables Simultaneous Reduction of Cardiac Ischemia-Reperfusion Injury. ACS Nano.

[52] Katsuki S., Matoba T., Nakashiro S., Sato K., Koga J.-I., Nakano K., Nakano Y., Egusa S., Sunagawa K., Egashira K. (2014). Nanoparticle-mediated delivery of pitavastatin inhibits atherosclerotic plaque destabilization/rupture in mice by regulating the recruitment of inflammatory monocytes. Circulation.

[53] Bakola V., Karagkiozaki V., Tsiapla A.R., Pappa F., Moutsios I., Pavlidou E., Logothetidis S. (2018). Dipyridamole-loaded biodegradable PLA nanoplatforms as coatings for cardiovascular stents. Nanotechnology.

[54] Wu X., Reboll M.R., Korf-Klingebiel M., Wollert K.C. (2020). Angiogenesis after acute myocardial infarction. Cardiovasc. Res..

[55] Quadros H.C., Ferreira Santos L.d.M., Meira C.S., Khouri M.I., Mattei B., Pereira Soares M.B., Castro-Borges W.D., Farias L.P., Formiga F.R. (2020). Development and in vitro characterization of polymeric nanoparticles containing recombinant adrenomedullin-2 intended for therapeutic angiogenesis. Int. J. Pharm..

[56] Shen J.-W., Li C., Yang M.-Y., Lin J.-F., Yin M.-D., Zou J.-J., Wu P.-Y., Chen L., Song L.-X., Shao J.-W. (2022). Biomimetic nanoparticles: U937 cell membranes based core–shell nanosystems for targeted atherosclerosis therapy. Int. J. Pharm..

[57] Ferreira M.P.A., Ranjan S., Kinnunen S., Correia A., Talman V., Mäkilä E., Barrios-Lopez B., Kemell M., Balasubramanian V., Salonen J. (2017). Drug-Loaded Multifunctional Nanoparticles Targeted to the Endocardial Layer of the Injured Heart Modulate Hypertrophic Signaling. Small.

[58] Liu S., Chen X., Bao L., Liu T., Yuan P., Yang X., Qiu X., Gooding J.J., Bai Y., Xiao J. (2020). Treatment of infarcted heart tissue via the capture and local delivery of circulating exosomes through antibody-conjugated magnetic nanoparticles. Nat. Biomed. Eng..

[59] Zamani P., Fereydouni N., Butler A.E., Navashenaq J.G., Sahebkar A. (2019). The therapeutic and diagnostic role of exosomes in cardiovascular diseases. Trends Cardiovasc. Med..

[60] Wu T., Liu W. (2022). Functional hydrogels for the treatment of myocardial infarction. NPG Asia Mater..

[61] Zhang Y., Zhu D., Wei Y., Wu Y., Cui W., Liuqin L., Fan G., Yang Q., Wang Z., Xu Z. (2019). A collagen hydrogel loaded with HDAC7-derived peptide promotes the regeneration of infarcted myocardium with functional improvement in a rodent mode. Acta Biomater..

[62] Wu T., Cui C., Huang Y., Liu Y., Fan C., Han X., Yang Y., Xu Z., Liu B., Fan G. (2020). Coadministration of an adhesive conductive hydrogel patch and an injectable hydrogel to treat MI. ACS Appl. Mater. Interfaces.

[63] Carlini A.S., Gaetani R., Braden R.L., Luo C., Christman K.L., Gianneschi N.C. (2019). Enzyme-responsive progelator cyclic peptides for minimally invasive delivery to the heart post-myocardial infarction. Nat. Commun..

[64] Peña B., Laughter M., Jett S., Rowland T.J., Taylor M.R.G., Mestroni L., Park D. (2018). Injectable Hydrogels for Cardiac Tissue Engineering. Macromol. Biosci..

[65] Liang S., Zhang Y., Wang H., Xu Z., Chen J., Bao R., Tan B., Cui Y., Fan G., Wang W. (2018). Paintable and Rapidly Bondable Conductive Hydrogels as Therapeutic Cardiac Patches. Adv. Mater..

[66] Kobayashi K., Ichihara Y., Tano N., Fields L., Murugesu N., Ito T., Ikebe C., Lewis F., Yashiro K., Shintani Y. (2018). Fibrin glue-aided, instant epicardial placement enhances the efficacy of mesenchymal stromal cell-based therapy for heart failure. Sci. Rep..

[67] Su T., Huang K., Daniele M.A., Hensley M.T., Young A.T., Tang J., Allen T.A., Vandergriff A.C., Erb P.D., Ligler F.S. (2018). Cardiac Stem Cell Patch Integrated with Microengineered Blood Vessels Promotes Cardiomyocyte Proliferation and Neovascularization after Acute Myocardial Infarction. ACS Appl. Mater. Interfaces.

[68] Hua C., Liu J., Hua X., Wang X. (2020). Synergistic Fabrication of Dose–Response Chitosan/Dextran/β-Glycerophosphate Injectable Hydrogel as Cell Delivery Carrier for Cardiac Healing After Acute Myocardial Infarction. Dose-Response.

[69] You Y., Kobayashi K., Colak B., Luo P., Cozens E., Fields L., Suzuki K., Gautrot J. (2021). Engineered cell-degradable poly(2-alkyl-2-oxazoline) hydrogel for epicardial placement of mesenchymal stem cells for myocardial repair. Biomaterials.

[70] Carrion C.C., Nasrollahzadeh M., Sajjadi M., Jaleh B., Jamalipour Soufi G., Iravani S. (2021). Lignin, lipid, protein, hyaluronic acid, starch, cellulose, gum, pectin, alginate and chitosan-based nanomaterials for cancer nanotherapy: Challenges and opportunities. Int. J. Biol. Macromol..

[71] Dasa K.S.S., Suzuki R., Gutknecht M., Brinton L.T., Tian Y., Michaelsson E., Lindfors L., Klibanov A.L., French B.A., Kelly K.A. (2015). Development of target-specific liposomes for delivering small molecule drugs after reperfused myocardial infarction. J. Control. Release.

[72] Li M., Tang X., Liu X., Cui X., Lian M., Zhao M., Peng H., Han X. (2020). Targeted miR-21 loaded liposomes for acute myocardial infarction. J. Mater. Chem. B.

[73] Sercombe L., Veerati T., Moheimani F., Wu S.Y., Sood A.K., Hua S. (2015). Advances and Challenges of Liposome Assisted Drug Delivery. Front. Pharmacol..

[74] Moosavian S.A., Bianconi V., Pirro M., Sahebkar A. (2021). Challenges and pitfalls in the development of liposomal delivery systems for cancer therapy. Semin. Cancer Biol..

[75] Santos L.M.F., Barreto B.C., Quadros H.C., Meira C.S., Carvalho R.d.S.F., Rebouças J.d.S., Macambira S.G., Vasconcelos J.F., Souza B.S.d.F., Soares M.B.P. (2022). Tissue response and retention of micro- and nanosized liposomes in infarcted mice myocardium after ultrasound-guided transthoracic injection. Eur. J. Pharm. Biopharm..

[76] Gothwal A., Kesharwani P., Gupta U., Khan I., Mohd Amin M.C.I., Banerjee S., Iyer A.K. (2015). Dendrimers as an Effective Nanocarrier in Cardiovascular Disease. Curr. Pharm. Des..

[77] Yu M., Jie X., Xu L., Chen C., Shen W., Cao Y., Lian G., Qi R. (2015). Recent Advances in Dendrimer Research for Cardiovascular Diseases. Biomacromolecules.

[78] Shen M., Yao S., Li S., Wu X., Liu S., Yang Q., Du J., Wang J., Zheng X., Li Y. (2021). A ROS and shear stress dual-sensitive bionic system with cross-linked dendrimers for atherosclerosis therapy. Nanoscale.

[79] Modi H.R., Wang Q., Olmstead S.J., Khoury E.S., Sah N., Guo Y., Gharibani P., Sharma R., Kannan R.M., Kannan S. (2022). Systemic administration of dendrimer N-acetyl cysteine improves outcomes and survival following cardiac arrest. Bioeng. Transl. Med..

[80] Fayed N.D., Goda A.E., Essa E.A., El Maghraby G.M. (2021). Chitosan-encapsulated niosomes for enhanced oral delivery of atorvastatin. J. Drug Deliv. Sci. Technol..

[81] Liu Q., Xu J., Liao K., Tang N. (2021). Oral Bioavailability Improvement of Tailored Rosuvastatin Loaded Niosomal Nanocarriers to Manage Ischemic Heart Disease: Optimization, Ex Vivo and In Vivo Studies. AAPS PharmSciTech.

[82] Ghasemiyeh P., Mohammadi-Samani S. (2018). Solid lipid nanoparticles and nanostructured lipid carriers as novel drug delivery systems: Applications, advantages and disadvantages. Res. Pharm. Sci..

[83] Gao Y., Gu W., Chen L., Xu Z., Li Y. (2008). The role of daidzein-loaded sterically stabilized solid lipid nanoparticles in therapy for cardio-cerebrovascular diseases. Biomaterials.

[84] Shrestha S.C., Ghebremeskel K., White K., Minelli C., Tewfik I., Thapa P., Tewfik S. (2021). Formulation and Characterization of Phytostanol Ester Solid Lipid Nanoparticles for the Management of Hypercholesterolemia: An ex vivo Study. Int. J. Nanomed..

[85] Purohit D., Jalwal P., Manchanda D., Saini S., Verma R., Kaushik D., Mittal V., Kumar M., Bhattacharya T., Rahman M.H. (2022). Nanocapsules: An Emerging Drug Delivery System. Recent Pat. Nanotechnol..

[86] Chaves P.d.S., Ourique A.F., Frank L.A., Pohlmann A.R., Guterres S.S., Ruver Beck R.C. (2017). Carvedilol-loaded nanocapsules: Mucoadhesive properties and permeability across the sublingual mucosa. Eur. J. Pharm. Biopharm..

[87] Molloy C.P., Yao Y., Kammoun H., Bonnard T., Hoefer T., Alt K., Tovar-Lopez F., Rosengarten G., Ramsland P.A., van der Meer A.D. (2017). Shear-sensitive nanocapsule drug release for site-specific inhibition of occlusive thrombus formation. J. Thromb. Haemost..

[88] Ribas J., Sadeghi H., Manbachi A., Leijten J., Brinegar K., Zhang Y.S., Ferreira L., Khademhosseini A. (2016). Cardiovascular organ-on-a-chip platforms for drug discovery and development. Appl. In Vitro Toxicol..

[89] Paloschi V., Sabater-Lleal M., Middelkamp H., Vivas A., Johansson S., van der Meer A., Tenje M., Maegdefessel L. (2021). Organ-on-a-chip technology: A novel approach to investigate cardiovascular diseases. Cardiovasc. Res..

[90] Rodrigues R.O., Sousa P.C., Gaspar J., Bañobre-López M., Lima R., Minas G. (2020). Organ-on-a-Chip: A Preclinical Microfluidic Platform for the Progress of Nanomedicine. Small.

[91] Cong Y., Han X., Wang Y., Chen Z., Lu Y., Liu T., Wu Z., Jin Y., Luo Y., Zhang X. (2020). Drug toxicity evaluation based on organ-on-a-chip technology: A review. Micromachines.

[92] Gonçalves I.M., Carvalho V., Rodrigues R.O., Pinho D., Teixeira S.F.C.F., Moita A., Hori T., Kaji H., Lima R., Minas G. (2022). Organ-on-a-chip platforms for drug screening and delivery in tumor cells: A systematic review. Cancers.

[93] Park J., Wu Z., Steiner P.R., Zhu B., Zhang J.X.J. (2022). Heart-on-chip for combined cellular dynamics measurements and computational modeling towards clinical applications. Ann. Biomed. Eng..

[94] Danku A.E., Dulf E.-H., Braicu C., Jurj A., Berindan-Neagoe I. (2022). Organ-On-A-Chip: A Survey of Technical Results and Problems. Front. Bioeng. Biotechnol..

[95] Qian F., Huang C., Lin Y.D., Ivanovskaya A.N., O’Hara T.J., Booth R.H., Creek C.J., Enright H.A., Soscia D.A., Belle A.M. (2017). Simultaneous electrical recording of cardiac electrophysiology and contraction on chip. Lab Chip.

[96] Soltantabar P., Calubaquib E.L., Mostafavi E., Ghazavi A., Stefan M.C. (2021). Heart/liver-on-a-chip as a model for the evaluation of cardiotoxicity induced by chemotherapies. Organs-on-a-Chip.

[97] Faulkner-Jones A., Zamora V., Hortigon-Vinagre M.P., Wang W., Ardron M., Smith G.L., Shu W. (2022). A Bioprinted Heart-on-a-Chip with Human Pluripotent Stem Cell-Derived Cardiomyocytes for Drug Evaluation. Bioengineering.

[98] Gonçalves I.M., Rodrigues R.O., Moita A.S., Hori T., Kaji H., Lima R.A., Minas G. (2022). Recent trends of biomaterials and biosensors for organ-on-chip platforms. Bioprinting.

[99] Pitingolo G., Nizard P., Riaud A., Taly V. (2018). Beyond the on/off chip trade-off: A reversibly sealed microfluidic platform for 3D tumor microtissue analysis. Sens. Actuators B Chem..

[100] Le N.X.T., Trinh K.T.L., Lee N.Y. (2021). Poly(acrylic acid) as an adhesion promoter for UV-assisted thermoplastic bonding: Application for the in vitro construction of human blood vessels. Mater. Sci. Eng. C.

[101] Park S.M., Kim H.M., Song K.H., Eom S., Park H.J., Doh J., Kim D.S. (2018). Ultra-thin, aligned, free-standing nanofiber membranes to recapitulate multi-layered blood vessel/tissue interface for leukocyte infiltration study. Biomaterials.

[102] Annabi N., Selimović Š., Acevedo Cox J.P., Ribas J., Bakooshli M.A., Heintze D., Weiss A.S., Cropek D., Khademhosseini A. (2013). Hydrogel-coated microfluidic channels for cardiomyocyte culture. Lab Chip.

[103] Lakshmanan R., Maulik N. (2018). Development of next generation cardiovascular therapeutics through bio-assisted nanotechnology. J. Biomed. Mater. Res. B Appl. Biomater..

[104] Fan C., Joshi J., Li F., Xu B., Khan M., Yang J., Zhu W. (2020). Nanoparticle-mediated drug delivery for treatment of ischemic heart disease. Front. Bioeng. Biotechnol..

[105] Ulbrich K., Holá K., Šubr V., Bakandritsos A., Tuček J., Zbořil R. (2016). Targeted Drug Delivery with Polymers and Magnetic Nanoparticles: Covalent and Noncovalent Approaches, Release Control, and Clinical Studies. Chem. Rev..

